# Increased Survival Concomitant with Unchanged Morbidity and Cognitive Disability among Infants Born at the Limit of Viability before 24 Gestational Weeks in 2009–2019

**DOI:** 10.3390/jcm12124048

**Published:** 2023-06-14

**Authors:** Yasemin Christiansson, Maria Moberg, Alexander Rakow, Ylva Vladic Stjernholm

**Affiliations:** 1 Karolinska University Hospital, Karolinska Institutet, Akademiska Stråket 14, 171 64 Stockholm, Sweden; yasemin.christiansson@capiostgoran.se (Y.C.); maria.moberg@stud.ki.se (M.M.); alexander.rakow@regionstockholm.se (A.R.); 2 Neonatal Unit, Department of Women’s and Children’s Health, Karolinska University Hospital, Karolinska Institutet, 171 77 Stockholm, Sweden; 3 Obstetric Unit, Department of Women’s and Children’s Health, Karolinska University Hospital, Karolinska Institutet, 171 76 Stockholm, Sweden

**Keywords:** cognition, fetal viability, language, morbidity, perinatal mortality, preterm birth

## Abstract

Introduction: The aim was to determine risk factors among mothers and outcomes for their children born at the limit of viability in 2009–2019, before and after the introduction of extended interventionist guidelines. Methods: A retrospective cohort study of births at 22 + 0–23 + 6 gestational weeks in a Swedish Region in 2009–2015 (n = 119), as compared to 2016–2019 (n = 86) after the introduction of new national interventionist guidelines. Infant mortality, morbidity, and cognitive functions at 2 years corrected age according to the Bayley-III Screening Test were monitored. Results: Maternal risk factors for extreme preterm birth were identified. The intrauterine fetal death rates were comparable. Among births at 22 weeks, the neonatal mortality tended to decrease (96 vs. 76% of live births (*p* = 0.05)), and the 2-year survival tended to increase (4 vs. 24% (*p* = 0.05)). Among births at 23 weeks, the neonatal mortality decreased (56 vs. 27% of live births (*p* = 0.01)), and the 2-year survival increased (42 vs. 64% (*p* = 0.03)). Somatic morbidity and cognitive disability at 2 years corrected age were unchanged. Conclusion: We identified maternal risk factors that emphasize the need for standardized follow-up and counseling for women at increased risk of preterm birth at the limit of viability. The increased infant survival concomitant with unchanged morbidity and cognitive disability highlight the importance of ethical considerations regarding interventionist approaches at threatening preterm birth before 24 weeks.

## 1. Introduction

The worldwide rate of all preterm birth (PTB) before 37 weeks GA (gestational age), the main cause of infant mortality up to 5 years of age, is still 10% [1]. Psychosocial stress, malnutrition, multiple pregnancy, short interpregnancy interval, choriodecidual bleeding, and intra-uterine inflammation are known risk factors for PTB [2,3,4,5,6,7]. Tocolytic treatments do not prevent PTB, but are given with an aim to delay delivery for at least 48 h to optimize the effect of antenatal corticosteroids for fetal lung maturation and to allow for transport to a tertiary hospital with Neonatal Intensive Care Unit (NICU) expertise [8,9]. A cervical length (CL) ≤ 25 mm in early pregnancy is regarded as a primary predictor for PTB [2,3,5,8,10]. Prophylactic progesterone treatment, which is recommended by the International Federation of Gynecology and Obstetrics (FIGO) for asymptomatic women with a previous PTB or a short CL, is effective according to some studies, except from those with the largest sample size [3,10].

Although the global survival rate of neonates born extremely preterm before 28 weeks GA has increased since the 1990s, a wide variety of recommendations exist, particularly between 22 and 24 weeks GA. These infants are at considerable risk of long term somatic morbidity such as growth restriction, cerebral palsy, visual and hearing loss, epileptic seizures, and neurodevelopmental impairment, such as poor language development, attention deficit disorders (ADHD), autism spectrum disorders, and intellectual disability [11,12,13,14,15,16,17,18].

One third of the 105–115,000 deliveries per year in Sweden take place in the Stockholm Region. The Karolinska University Hospital Solna is a tertiary referral center for extreme PTB at the limit of viability in the Stockholm Region [19]. Among the 3500 childbirths per year at the hospital, approximately 60 infants are born before 28 + 0 weeks GA, and 100 infants before 34 + 0 weeks. The NICU provides treatment with continuous positive airway pressure (CPAP), nasal high flow, respirator care, and extracorporeal membrane oxygenation (ECMO) care. The Swedish definition of PTB is birth between 22 + 0 and 36 + 6 weeks GA, in accordance with the World Health Organization definition [20].

### Aim

The aim of this study was to determine maternal risk factors and infant mortality and morbidity at 2 years corrected age in children born before 24 gestational weeks in 2009–2019. We hypothesized that the introduction of new interventionist guidelines would have resulted in improved infant survival, reduced morbidity, and improved cognitive functions.

## 2. Material and Methods

Study design. This study is a retrospective single center cohort study of mothers and their children born before 24 weeks GA in the Stockholm Region in the years 2009–2019, before and after the introduction of new national interventionist guidelines. In 2009–2015, n = 119 infants were born before 24 weeks GA. According to clinical guidelines in these years, predelivery transport to a tertiary level hospital with NICU expertise was recommended at 22 + 5 weeks; antenatal corticosteroids and neonatal resuscitation were considered from 22 + 5 weeks and recommended from 24 + 0 weeks. In 2016–2019, n = 86 infants were born before 24 weeks GA. In 2016, the Swedish Neonatal Society and the perinatal group within the Swedish Society of Obstetricians and Gynecologists, together with the heads of the neonatology and obstetrics units at Swedish university hospitals, agreed on consensus guidelines for the management of those who are at the border of viability. The consensus was mainly based on the data from the EXPRESS study, together with recent data from the Swedish Neonatal Quality Register (SNQ) [21]. According to new guidelines, predelivery transport to a tertiary hospital with NICU expertise was recommended at 22 + 0 weeks; antenatal corticosteroids and neonatal resuscitation were considered from 22 + 0 weeks and recommended from 23 + 0 weeks [21]. Ethics approval for maternal data collection was obtained from the Regional Ethics Board for Medical Sciences in Stockholm 9 April 2015, No 2014/255-31, and ethics approval for infant data collection was obtained from the Ethics Board of Medical Sciences 11 March 2020, No 2019/06576. Since all data were retrieved in retrospect and presented on a group basis only, without any possibility to trace any personal information, individual informed consent from participants was not required from the Regional Ethics Board for Medical Sciences in Stockholm. All data were retrieved from original electronic medical records (Obstetrix^®^ Cerner AB, Stockholm, Sweden, and TakeCare Hospital Management Software System).

Maternal data. Age, body mass index (BMI), parity, previous PTB or cervical conization, cervical cerclage, intercurrent diseases, pregnancy complications, and primary reasons behind the PTB were documented. According to clinical guidelines, 2 doses of antenatal betamethasone, 12 mg, were given intramuscularly with a 12–24 h interval for fetal lung maturation from 22 + 5 weeks in 2009–2015 and from 22 + 0 weeks in 2016–2019. In all years studied, prophylactic oral erytromycin was given at preterm prelabor rupture of the fetal membranes (pPROM), and prophylactic intravenous bensylpenicillin, 3 g, was given every 6 h during active labor before 37 weeks.

Infant data. GA, birth weight (BW), Apgar score < 7 at 5 min, intrauterine fetal death (IUFD) rate, neonatal mortality ≤ 28 days, and 2-year survival were registered. Live birth was defined as an infant with any signs of life at birth [14]. IUFD was deaths delivered at ≥22 + 0 weeks GA, according to the WHO definition [20].

Neonatal respiratory distress syndrome (RDS), bronchopulmonary dysplasia (BPD), persistent pulmonary hypertension (PPH), intraventricular hemorrhage (IVH), neonatal seizures, sepsis or pneumonia, necrotizing enterocolitis (NEC), abdominal surgery, retinopathy of prematurity (ROP), persistent ductus arteriosus (DA), hyperbilirubinemia, and anemia were monitored. Neonatal RDS was defined by clinical diagnosis of type I RDS and a requirement of oxygen therapy for at least 24 h, according to the World Health Organization classification [22]. At 2 years of corrected age, certified psychologists assessed cognitive, language, and motor development with the Bayley Scales of Infant and Toddler Development 3rd Ed, which are standardized to mean ± SD scores of 100 ± 15 [23]. Cognitive, motor, and language development were considered normal if the composite score on the respective Bayley-III Scale was ≥mean − 1SD, mildly impaired if the score was less than mean − 1SD but ≥mean − 2SD, moderately impaired if the score was less than mean − 2SD but ≥mean − 3SD, and severely impaired if the score was mean less than − 3SD [14].

Statistical analysis. Continuous data were analyzed using the Mann–Whitney U-test, and were presented as mean ± standard deviation (SD) or median and interquartile range (IQR). Categorical data were analyzed with Chi^2^-test and Fisher’s exact test when appropriate, and were presented as numbers and percentages. A *p* value < 0.05 was considered significant.

## 3. Results

**Figure 1:**
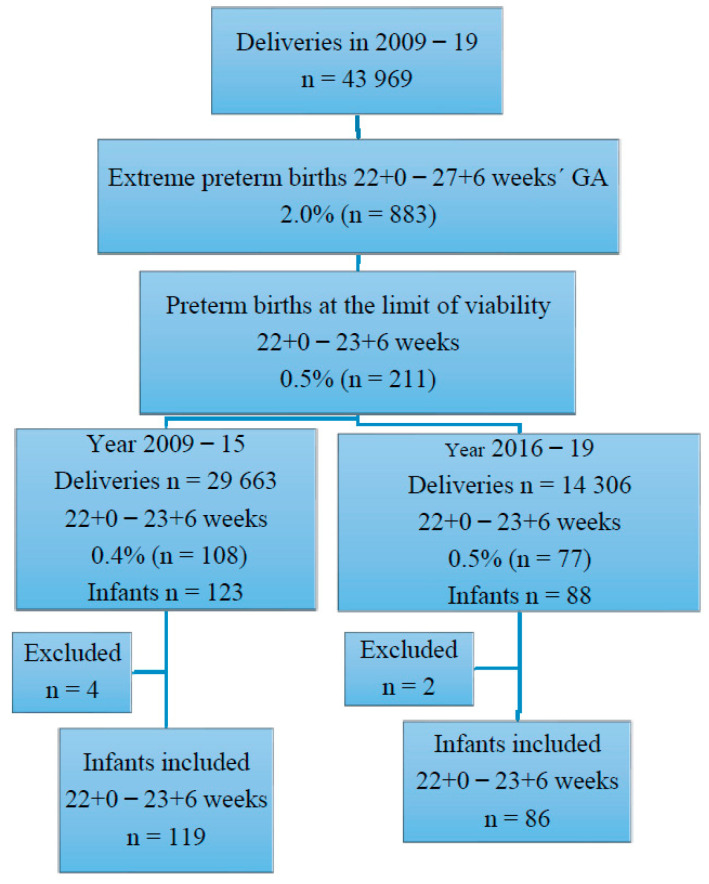
Inclusion of mothers and their infants born before 24 weeks GA in 2009–2019. Neonates in 2009–2015 (n = 4) and 2016–2019 (n = 2), who were transferred to other regions within a few weeks after birth, were excluded from the calculations.

A flow chart over mother and infant inclusion is shown in Figure 1.

Maternal outcomes. Maternal characteristics are shown in Table 1. Maternal characteristics were comparable between the groups, except for the rate of diabetes during pregnancy, which increased from 1 to 9% (*p* = 0.04), and which was observed after altered national diagnostic criteria in 2017. According to these, gestational diabetes is diagnosed after a fasting blood glucose level of ≥5.1 mmol/L, or a 2 h value of ≥8.5 mmol/L after a 75 g oral glucose loading tolerance test (OGTT). The rate of women who received 2 doses of antenatal steroids increased from 32 to 55% (*p* = 0.002). The main reason behind a PTB was preterm labor (PTL) in both groups, followed by preterm prelabor rupture of the fetal membranes (pPROM) in 31 vs. 17% (*p* = 0.03). Clinical chorioamnionitis was diagnosed in 32 vs. 36% (*p* = 0.46). The majority of women went through a vaginal delivery. The cesarean section rate was 15 %, and increased with advancing GA. The majority > 75% of the births were spontaneous and <25% were induced due to fetal or maternal reasons.

Infant outcomes. Infant mortality is shown in Table 2, Table 3 and Table 4. For all infants taken together, the IUFD rates were comparable, the neonatal mortality decreased, and the 2-year survival increased to 51% of live births (Table 2). Among infants born at 22 weeks (Table 3), the neonatal mortality tended to decrease, and the 2-year survival tended to increase to 24% of live births (*p* = 0.05). At 23 weeks (Table 4), the neonatal mortality decreased, and the 2-year survival increased to 64% of live births (*p* = 0.03).

Infant morbidity is shown in Table 5. The composite morbidity rates were comparable between the study periods, except for ROP stage 1–5, which increased from 68 to 97%, and severe ROP stage 4–5, including partial ablatio retinae or total ablatio retinae with blindness, which increased from 32 to 47 % (*p* = 0.02).

Cognitive and language functions at 2 years corrected age are shown in Table 6. The Bayley-III Scale for assessment was applied for 52% of surviving infants in 2009–2015 and for 82% in 2016–2019. No cognitive or language disability was observed in 15% (2/13) vs. 7% (2/28) of infants (data not shown). The mean cognition index scores (87 vs. 83) were comparable between the study periods. Moderate or severe cognitive disability with a score of ≤82 was observed in 4 of 10 infants in both groups. Likewise, the mean language index scores (80 vs. 75) were comparable. Moderate or severe language disability with a score of ≤84 was observed in 6–7 of 10 infants. Motor function scores were not included in the results, due to few observations.

## 4. Discussion

We have determined maternal risk factors, proportions of IUFD, infant mortality ≤ 28 days, 1- and 2-year survival, somatic morbidity, and cognitive functions at 2 years corrected age among children born at the limit of viability before 24 weeks GA during 2009–2019 in a Swedish region, before and after the introduction of extended interventionist guidelines. We hypothesized that the new guidelines would have resulted in increased infant survival, reduced morbidity, and improved cognitive functions. Our results showed increased infant survival concomitant with unchanged somatic morbidity and cognitive disability. Our hypothesis was therefore rejected.

Maternal characteristics did not differ, except for a higher rate of diabetes during pregnancy in the latter period due to altered diagnostic criteria (Table 1). Although the maternal characteristics in the study groups were comparable, some variables differed from the general population of women of reproductive age [2]. More mothers in the study groups had a medical history of a previous IUFD, cervical conization or PTB, a multiple pregnancy, IVF pregnancy, cervical cerclage, placental abruption, or a clinical chorioamnionitis [2,24,25,26,27]. The high rate of clinical chorioamnionitis in the study groups was in accordance with previous reports on uterine inflammation, which is sometimes related to the presence of low virulent pathogens such as *Mycoplasma and Ureaplasma* sp., *anaerobes*, *group B Streptococci*, or *E. coli* in 30–60% of cases and histological chorioamnionitis in more than 90% of all PTB before 24 weeks [2,27,28,29]. Physiological changes of the vaginal microbiome in pregnant women result in a lower pH than in the non-pregnant state, and failure to develop such physiological alterations is associated with PTB [6,7,29]. It was not an aim of this study to investigate the psychosocial wellbeing among the parents. Several authors report that children born very preterm are at increased risk of experiencing parental or caregiver changes and instability, with the greatest risk for those born extremely preterm. Preterm birth and exposure to parental or caregiver instability may contribute additively to poorer child outcomes [30,31].

Our results for infant outcomes showed that the proportions of IUFD were comparable between the study periods. The 2-year survival among infants born at 22 weeks tended to increase from 4 to 24% of live births, and the 2-year survival at 23 weeks increased from 42 to 64%. The present results were in agreement with the Swedish multicenter EXPRESS study of extremely preterm infants in 2004–2007, which reported a 1-year survival of 10% (5/51) of live births at 22 weeks and 52% (53/101) at 23 weeks [14]. The British multicenter EPICure study of extremely preterm infants in 2006 reported a 3-year survival of 2% (3/152) of live births at 22 weeks and 18% (63/339) at 23 weeks [12]. The Japanese Neonatal Network study of extremely preterm infants, including tertiary centers only, in 2003–2005 reported a 3-year survival of 36% (27/75) at 22 weeks and 63% (154/245) at 23 weeks [13].

The present results showed that the composite somatic morbidity at 2 years corrected age did not differ between the study periods, except for the proportion of ROP, which was actually higher in the latter period (Table 5).

We found that the cognitive functions at 2 years corrected age, assessed according to the Bayley-III Screening Test, were comparable between the study periods (Table 6). The mean cognition index scores and mean language index scores were comparable. In both periods, moderate or severe cognitive disability was observed in 4 of 10 infants, and moderate or severe language disability in 6–7 of 10 infants. The present results should be interpreted with caution due to the limited number of observations. Nevertheless, they were in accordance with a Swedish register study of extremely preterm infants born in 2007–2018, where neurodevelopmental diagnoses were investigated at 6 years of age. The register data showed that, for children born at 22 weeks (n = 93), 78% had any neurodevelopmental disorder, 64% were referred to habilitation services, 49% had intellectual disability, 28% had autism spectrum disorders, 25% had ADHD, 29% had visual impairment, 6% had hearing disorders, and 56% had speech disorders. Among children born at 23 weeks (n = 290), 74% had any neurodevelopmental disorder, 52% were referred to habilitation services, 36% had intellectual disability, 23% had autism spectrum disorders, 32% had ADHD, 19% had visual impairment, 5% had hearing impairment, and 51% had speech disorders [18].

Our results were in agreement with previous reports of no major improvements in cognitive functions among infants born before 24 weeks since the 1990s [12,15,16,17,18]. The British multicenter EPICure study reported unchanged cognitive functions at 2 years of age among children born before 24 weeks between 1995 and 2006 [12]. The Japanese Neonatal Network study reported no or minimally impaired cognitive functions in 12% (9/75) of infants born at 22 weeks and in 20% (49/245) of those born at 23 weeks in 2003–2005 [13].

The present observations and previous research [11,12,13,14,15,16,17,18] raise several ethical concerns regarding the justification of interventions and life-sustaining treatments for threatening PTB before 24 weeks. The interventionist approach in Sweden and other Nordic countries contrast to the more restrictive approaches in other parts of the world [21,32,33]. A systematic review of guidelines for extremely preterm deliveries between 22 and 25 weeks in 20 highly developed countries finds a general agreement for comfort care at 22 weeks and active care at 25 weeks, but a wide variety of recommendations between 23 and 24 weeks [34]. National guidelines in the Netherlands recommend resuscitation of all newborns only at 24 weeks and thereafter, based on results from large national studies The ethical justification for this restrictive practice is that the amount of good, which is less than 10% intact survival, does not justify the amount of harm caused, with 10–20% disabilities and 70–80% mortality after extensive treatments [32,33].

The strength of our study was that all data were collected from original medical records at one hospital. All data were retrieved by a limited group of investigators, and a subset of original records were re-investigated to assure accuracy. Biases due to different clinical practices and transfer of clinical data to registers were therefore avoided. Another strength was that IUFD and extreme PTB at the limit of viability were clearly defined and determined. Limitations were the retrospective character, the limited number of observations, and the limited follow-up until 2 years of age. The results must therefore be interpreted with caution. Moreover, although the Bayley Screening Test is widely used, it is still only a modest approach with limited value to describe cognitive functions adequately. Nevertheless, one third of all births in Sweden occur in the Stockholm Region, and the present results were in accordance with national register data on neurodevelopmental diagnoses at 6 years of age [18]. Moreover, it is suggested that early infant cognitive impairment after extreme PTB may persist unto adult life [15].

## 5. Conclusions

We identified maternal risk factors that emphasize the need of standardized follow-up and counseling for women at increased risk of preterm birth at the limit of viability. The increased infant survival concomitant with unchanged morbidity and cognitive disability highlight the importance of ethical considerations regarding interventionist approaches for threatening preterm birth before 24 weeks.

## Figures and Tables

**Table 1:** Maternal characteristics. Statistical methods: Mann–Whitney U-test with General Linear Model when appropriate ^1^, Chi2-test and Fisher’s exact test when appropriate ^2^, Kruskal–Wallis test ^3^.

Variable	2009–2015 n = 108	2016–2019 n = 77	*p* Value
Age, year, (median ± IQR)	31 (27–35)	32 (28–35)	0.81 ^1^
BMI, kg/m^2^, (median ± IQR)	26 (22–29)	25 (22–27)	0.78 ^1^
Primiparous, n (%)	66 (61)	47 (61)	0.99 ^2^
Previous PTB, n (%)	28 (26)	21 (27)	0.84 ^2^
Pregnancy, n (%)			0.70 ^3^
Singleton	96 (89)	67 (87)	
Duplex	10 (8)	10 (13)	
Triplex	1 (1)	0	
Quadriplex	1 (1)	0	
In vitro fertilization, n (%)	18 (17)	11 (14)	0.62 ^2^
Cervical conisation, n (%)	8 (7)	8 (10)	0.48 ^2^
Cervical cerclage, n (%)	12 (11)	8 (10)	0.88 ^2^
Hypertensive disease, n (%)	13 (12)	6 (8)	0.35 ^2^
Diabetes, n (%)	1 (1)	7 (9)	0.04 ^2^
Main reason for PTB n (%)			0.03 ^3^
Preterm labor	53 (49)	50 (65)	
pPROM	33 (31)	13 (17)	
IUGR with signs of fetal asphyxia	9 (8)	5 (6)	
Hypertensive disease	3 (3)	4 (5)	
Maternal disease	1 (1)	0	
Choriodecidual bleeding, n (%)	12 (11)	11 (14)	0.52 ^2^
Clinical chorioamnionitis, n (%)	35 (32)	29 (36)	0.46 ^2^
Antenatal corticosteroids, n (%)			0.002 ^3^
None	51 (48)	22 (29)	
1 dose	22 (21)	13 (17)	
2 doses	34 (32)	42 (55)	
Delivery mode, n (%)			0.76 ^3^
Vaginal delivery	92 (85)	66 (86)	
Cesarean section	16 (15)	11 (14)	

Abbreviations: BMI = body mass index, IQR = interquartile range, IUGR = Intrauterine growth restriction, PTB = preterm birth, pPROM = preterm prelabor rupture of fetal membranes, SD = standard deviation.

**Table 2:** Survival of infants born before 24 weeks GA. Statistical methods: Mann–Whitney U-test with General Linear Model when appropriate ^1^, Chi2-test and Fisher’s exact test when appropriate ^2^.

Variable	2009–2015 n = 119	2016–2019 n = 86	*p* Value
GA, n (%)			0.74 ^2^
22 + 0–6 wks	43 (36)	33 (38)	
23 + 0–6 wks	76 (64)	53 (62)	
Gender, n (%)			0.99 ^2^
Female	53 (45)	39 (45)	
Male	66 (55)	47 (55)	
BW, g (median ± IQR)	526 (459–590)	517 (462–589)	0.81 ^1^
Apgar score < 7, n (%)	100/119 (84)	75/86 (87)	0.11 ^2^
Live births, n (%)	81/119 (68)	66/86 (77)	0.12 ^2^
Intrauterine fetal death, n (%)	38/119 (32)	20/86 (23)	0.12 ^2^
Neonatal death ≤ 28 days, n (%)			
Of all births	50/119 (42)	25/86 (29)	0.01 ^2^
Of live births	50/81 (62)	25/66 (38)	0.01 ^2^
1- and 2-year survival, n (%)			
Of all births	25/119 (21)	34/86 (39)	0.004 ^2^
Of live births	25/81 (31)	34/66 (51)	0.01 ^2^

**Table 3:** Survival of infants born at 22 weeks GA. Statistical method: Chi2-test and Fisher’s exact test when appropriate.

Variable	2009–2015 n = 43 (%)	2016–2019 n = 33 (%)	*p* Value
Live births	24/43 (56)	21/33 (64)	0.55
Intrauterine fetal death	19/43 (44)	12/33 (36)	0.55
Neonatal death ≤ 28 days			
Of all births	23/43 (53)	16/33 (48)	0.18
Of live births	23/24 (96)	16/21 (76)	0.05
1- and 2-year survival			
Of all births	1/43 (2)	5/33 (15)	0.04
Of live births	1/24 (4)	5/21 (24)	0.05

**Table 4:** Survival of infants born at 23 weeks GA. Statistical method: Chi2-test and Fisher’s exact test when appropriate.

Variable	2009–2015 n = 76 (%)	2016–2019 n = 53 (%)	*p* Value
Live births	57/76 (75)	45/53 (85)	0.16
Intrauterine fetal death	19/76 (25)	8/53 (20)	0.16
Neonatal death ≤ 28 days			
Of all births	32/76 (42)	12/53 (17)	0.005
Of live births	32/57 (56)	12/45 (27)	0.01
1- and 2-year survival			
Of all births	24/76 (32)	29/53 (55)	0.01
Of live births	24/57 (42)	29/45 (64)	0.03

Abbreviations: BW = birth weight, GA = gestational age, n = number of observations.

**Table 5:** Infant morbidity at 2 years corrected age. Statistical method: Chi2-test and Fisher’s exact test when appropriate ^1^, and Kruskal–Wallis test ^2^.

Variable	2009–2015 n = 25 (%)	2016–2019 n = 34 (%)	*p* Value 2 Sided Exact
GA			0.29 ^1^
22 + 0–6 wks	1 (4)	5 (15)	
23 + 0–6 wks	24 (96)	29 (85)	
Gender			0.83 ^2^
Female	11 (44)	14 (41)	
Male	14 (56)	20 (59)	
Pregnancy			0.65 ^2^
Singleton	19 (76)	24 (71)	
Twin	6 (24)	10 (29)	
Antenatal steroids			0.08 ^2^
None	5 (20)	1 (3)	
1 dose	5 (20)	8 (23)	
2 doses	15 (60)	25 (74)	
RDS	24 (96)	32 (94)	0.75 ^1^
BPD	22 (88)	28 (85)	0.58 ^1^
PPH	4 (16)	6 (18)	0.87 ^1^
IVH stage 1–4	10 (40)	15 (45)	0.82 ^1^
IVH severe stage 3–4	3 (12)	3 (9)	
Neonatal seizures	1 (4)	1 (3)	0.83 ^1^
Sepsis or pneumonia	18 (72)	26 (76)	0.70 ^1^
NEC diagnosis	7 (28)	6 (18)	0.64 ^2^
Ileus	1 (4)	2 (6)	
Bowel perforation	0	1 (3)	
Abdominal surgery	5 (25)	8 (24)	0.56 ^1^
ROP stage 1–5	17 (68)	33 (97)	0.02 ^1^
ROP severe stage 4–5	8 (32)	16 (47)	
Persistent ductus arteriosus	23 (92)	29 (85)	0.44 ^1^
Hyperbilirubinemia	20 (80)	31 (91)	0.22 ^1^
Anemia	22 (88)	30 (88)	0.98 ^1^

Abbreviations: BPD = bronchopulmonary dysplasia, IVH = intraventricular hemorrhage, n = number of observations, NEC = necrotizing enterocolitis, PPH = persistent pulmonary hypertension of the newborn, RDS = respiratory distress syndrome, ROP = retinopathy of prematurity.

**Table 6:** Cognitive functions according to Bayley-III Scale assessment at 2 years corrected age. Statistical method: Mann–Whitney U-test with General Linear Model when appropriate ^1^, Chi2-test and Fisher’s exact test when appropriate ^2^.

Variable	2009–2015 n = 25	2016–2019 n = 34	*p* Value	2009–2015 22 wks	2016–2019 22 wks	2009–2015 23 wks	2016–2019 23 wks
GA, n (%)				1	5	24	29
22 + 0–22 + 6 wks	1 (4)	5 (15)					
23 + 0–23 + 6 wks	24 (96)	29 (85)					
Bayley-III Scale, n (%)	13/25 (52)	28/34 (82)	0.01 ^2^	1 (100)	5 (100)	12 (50)	23 (79)
Cognition index score, mean (95% CI)	86.9 (14.0–32.2)	82.7 (14.2–24.7)	0.33 ^1^				
Cognition index score, n (%)	n = 13	n = 28					
Moderate/severe disability (score ≤ 82)	5 (38)	12 (43)		0	5	5	7
No/mild disability (score ≥ 83)	8 (62)	16 (57)		1	0	7	16
Language index score, mean (95% CI)	79.6 (13.3–32.3)	75.0 (17.3–30.1)	0.39 ^1^				
Language index score, n (%)	n = 13	n = 28					
Moderate/severe disability (score ≤ 84)	8 (62)	21 (75)		0	3	8	18
No/mild disability (score ≥ 85)	5 (38)	7 (25)		1	2	4	5

Abbreviations: CI = confidence interval, GA = gestational age, n = number of observations.

## Data Availability

All data analyzed during this trial are included in this article and will be provided by the corresponding author if requested.

## Abbreviations

BW, Birth Weight; CL, Cervical Length; IUFD Intrauterine Fetal Death; IUGR, Intrauterine Growth Restriction; IVH, Intraventricular Hemorrhage; IQR, Interquartile Range; GA, Gestational Age; NEC, Necrotizing Enterocolitis; NICU, Neonatal Intensive Care Unit; PTB, Preterm Birth, PTL, Preterm Labor, RDS, Respiratory Distress Syndrome; ROP, Retinopathy of Prematurity. ECMO = extracorporeal membrane oxygenation.
